# Incidental radiological findings during clinical tuberculosis screening in Lesotho and South Africa: a case series

**DOI:** 10.1186/s13256-023-04097-4

**Published:** 2023-08-25

**Authors:** Naomi Glaser, Shannon Bosman, Thandanani Madonsela, Alastair van Heerden, Kamele Mashaete, Bulemba Katende, Irene Ayakaka, Keelin Murphy, Aita Signorell, Lutgarde Lynen, Jens Bremerich, Klaus Reither

**Affiliations:** 1 Faculty of Medicine, University of Zürich, Zurich, Switzerland; 2 Department of Health Sciences and Medicine, University of Lucerne, Lucerne, Switzerland; 3 Center for Community Based Research, Human Sciences Research Council, Pietermaritzburg, South Africa; 4 SolidarMed, Partnerships for Health, Maseru, Lesotho; 5 Radboud University Medical Center, Nijmegen, The Netherlands; 6 Swiss Tropical and Public Health Institute, Allschwil, Switzerland; 7 University of Basel, Basel, Switzerland; 8 Institute of Tropical Medicine Antwerp, Antwerp, Belgium; 9 Department of Radiology, Clinic of Radiology and Nuclear Medicine, University Hospital Basel, University of Basel, Basel, Switzerland

**Keywords:** Case series, Chest X-ray, Non-TB abnormalities, CAD4TB, Sub-Saharan Africa

## Abstract

**Background:**

Chest X-ray offers high sensitivity and acceptable specificity as a tuberculosis screening tool, but in areas with a high burden of tuberculosis, there is often a lack of radiological expertise to interpret chest X-ray. Computer-aided detection systems based on artificial intelligence are therefore increasingly used to screen for tuberculosis-related abnormalities on digital chest radiographies. The CAD4TB software has previously been shown to demonstrate high sensitivity for chest X-ray tuberculosis-related abnormalities, but it is not yet calibrated for the detection of non-tuberculosis abnormalities. When screening for tuberculosis, users of computer-aided detection need to be aware that other chest pathologies are likely to be as prevalent as, or more prevalent than, active tuberculosis. However, non­-tuberculosis chest X-ray abnormalities detected during chest X-ray screening for tuberculosis remain poorly characterized in the sub-Saharan African setting, with only minimal literature.

**Case presentation:**

In this case series, we report on four cases with non-tuberculosis abnormalities detected on CXR in TB TRIAGE + ACCURACY (ClinicalTrials.gov Identifier: NCT04666311), a study in adult presumptive tuberculosis cases at health facilities in Lesotho and South Africa to determine the diagnostic accuracy of two potential tuberculosis triage tests: computer-aided detection (CAD4TB v7, Delft, the Netherlands) and C-reactive protein (Alere Afinion, USA). The four Black African participants presented with the following chest X-ray abnormalities: a 59-year-old woman with pulmonary arteriovenous malformation, a 28-year-old man with pneumothorax, a 20-year-old man with massive bronchiectasis, and a 47-year-old woman with aspergilloma.

**Conclusions:**

Solely using chest X-ray computer-aided detection systems based on artificial intelligence as a tuberculosis screening strategy in sub-Saharan Africa comes with benefits, but also risks. Due to the limitation of CAD4TB for non-tuberculosis-abnormality identification, the computer-aided detection software may miss significant chest X-ray abnormalities that require treatment, as exemplified in our four cases. Increased data collection, characterization of non-tuberculosis anomalies and research on the implications of these diseases for individuals and health systems in sub-Saharan Africa is needed to help improve existing artificial intelligence software programs and their use in countries with high tuberculosis burden.

## Introduction

Tuberculosis (TB) is a communicable disease that is a major cause of morbidity and among the leading causes of death worldwide [1]. To reach the World Health Organization (WHO)’s goal of reducing TB deaths by 95% by 2035, efficient screening strategies need to be implemented [2]. These strategies should include community-based active case finding programs, especially in areas with a high burden of disease where access to diagnostics in health facilities is restricted [3, 4]. Increasing evidence has shown that half of all microbiologically confirmed TB cases are subclinical, that is, asymptomatic (absence of cough, fever, night sweats, and weight loss), and can therefore be identified only through active screening rather than passive case finding [5]. This high proportion of subclinical TB cases has also been confirmed in TB prevalence surveys conducted in Lesotho and South Africa (SA) [6–8] and is one of the main reasons why the WHO recommends a symptom-agnostic, community-based screening approach with chest X-ray (CXR) to close the TB detection gap [5]. CXR offers high sensitivity and acceptable specificity as a TB screening tool [9]; however, specialist opinion and radiological expertise for CXR interpretation is often lacking in high-burden, low-resource settings [10–12]. Computer-aided detection (CAD) software using artificial intelligence (AI) offers a reliable solution to perform and accelerate active case findings in such settings, allowing detection of TB-related abnormalities in CXR without the need for an on-site radiologist [13]. CAD4TB (Delft Imaging Systems, NL) is a digital CXR analysis software which produces an abnormality score between 0 and 100, increasing with TB-associated abnormality, thus reflecting the probability of active TB visible on the CXR [14, 15]. Besides the score, the CAD4TB algorithm also outputs a heatmap, which visually indicates abnormal areas in the lung fields [16]. The use of CAD for triage requires a context-specific threshold at which participants are referred for confirmatory TB testing. This threshold should be established for each setting and target population [6]. At health facilities in Lesotho and SA, we conducted the TB TRIAGE + ACCURACY study in adult presumptive TB cases to determine the diagnostic accuracy of two potential TB triage tests, namely CAD (CAD4TB v7, Delft, the Netherlands) and C-reactive protein (CRP; Alere Afinion, USA) to be used in a second large-scale collaborative study on novel TB case detection strategies [16]. Expectorated sputum samples for reference standard were collected as follows: The first sputum sample was equally split in two aliquots to be tested on-site with Xpert MTB/RIF and Xpert MTB/RIF Ultra (Cepheid, Sunnyvale, USA), respectively. The second sputum sample was used for Bactec MGIT 960 culture (Becton, Dickinson and Co., Franklin Lakes, USA). Additionally, participants received human immunodeficiency virus (HIV) testing according to national guidelines and an advanced HIV disease care package including VISITECT CD4 (Omega Diagnostics, UK) and collection of a urine sample from people living with HIV for a Determine^TM^ TB Lipoarabinomannan (LAM) Ag test (AlereLAM; Abott Diagnostics, Chicago, USA). Digital chest radiographs were analyzed by the CAD4TB software and by a human expert radiologist at the University Hospital Basel, Switzerland, via secure teleradiology. In this case series, we report on four non-TB cases that were detected as non-TB abnormalities in TB TRIAGE + ACCURACY. Using this exemplary case series, we will discuss the risks and benefits of using solely CAD as a TB screening strategy in sub-Saharan Africa.

## Case presentations

### Case I

**Fig. 1:**
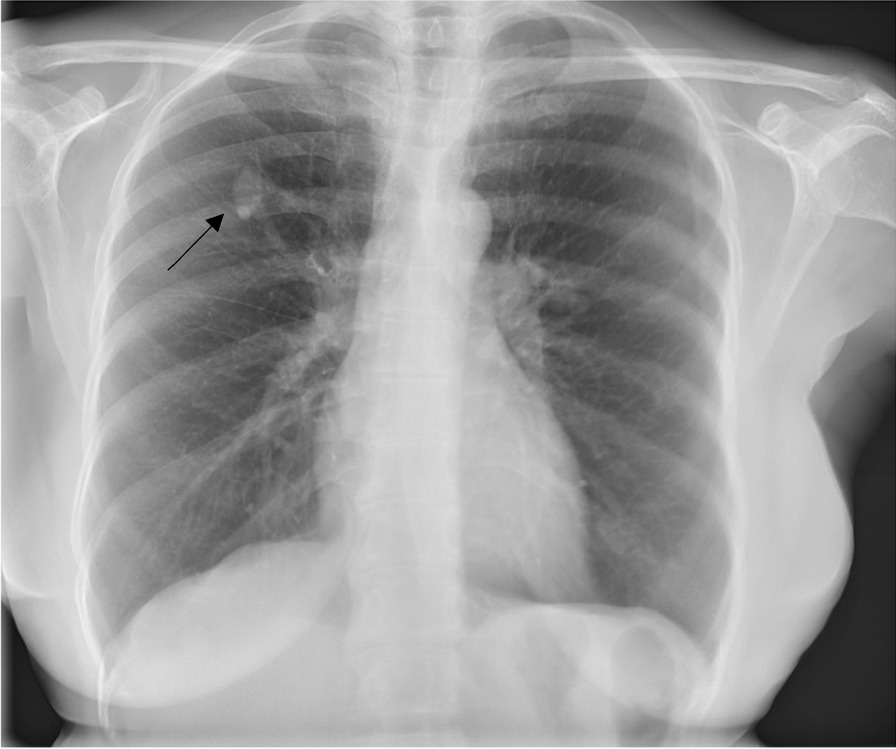
Arrow pointing to the AV malformation in the right upper lobe with feeding/draining vessel

A 59-year-old African woman living with HIV reported for TB screening in Butha-Buthe District Hospital, Lesotho with a cough, sputum production, and chest pain that began 2 weeks prior to presentation. Additionally, she reported rhinorrhea, which started 1 week later. No fever, weight loss, night sweats, fatigue, headache, or hemoptysis was reported. She had been on antiretroviral therapy (ART) since 2009 with a good adherence, currently on co-formulated tenofovir disoproxil fumarate (TDF) + lamivudine (3TC) + dolutegravir (DTG). Previous history of TB, other lung diseases, or other comorbidities was not reported by the participant. On clinical assessment, she was afebrile and her vital parameters, including oxygen saturation, were all within normal range. Further physical examination was unremarkable. The severe acute respiratory syndrome coronavirus 2 (SARS-CoV-2) polymerase chain reaction (PCR), Xpert MTB/RIF, and Xpert MTB/RIF Ultra assays, as well as the Determine TB LAM Ag test all returned negative results. The semi-quantitative VISITECT CD4 test showed a CD4 level above 200 cells/µL. The posterior–anterior CXR (PA-CXR), which was interpreted by an expert radiologist at the University Hospital in Basel, revealed an opacity in the right upper lobe, highly suspicious for a pulmonary arteriovenous malformation (PAVM) with a feeding pulmonary artery and a draining pulmonary vein (Fig. 1; CAD4TB v7 score: 15.86). After the initial presentation and enrollment in the study, the patient sought no further treatment because her cough subsided. In a follow-up interview, she stated that the finding on the CXR taken in the study was explained to her; however, she only recollected that there was no clinical significance of TB or coronavirus disease 2019 (COVID-19), suggesting that she was never clearly informed concerning her PAVM.

### Case II

**Fig. 2:**
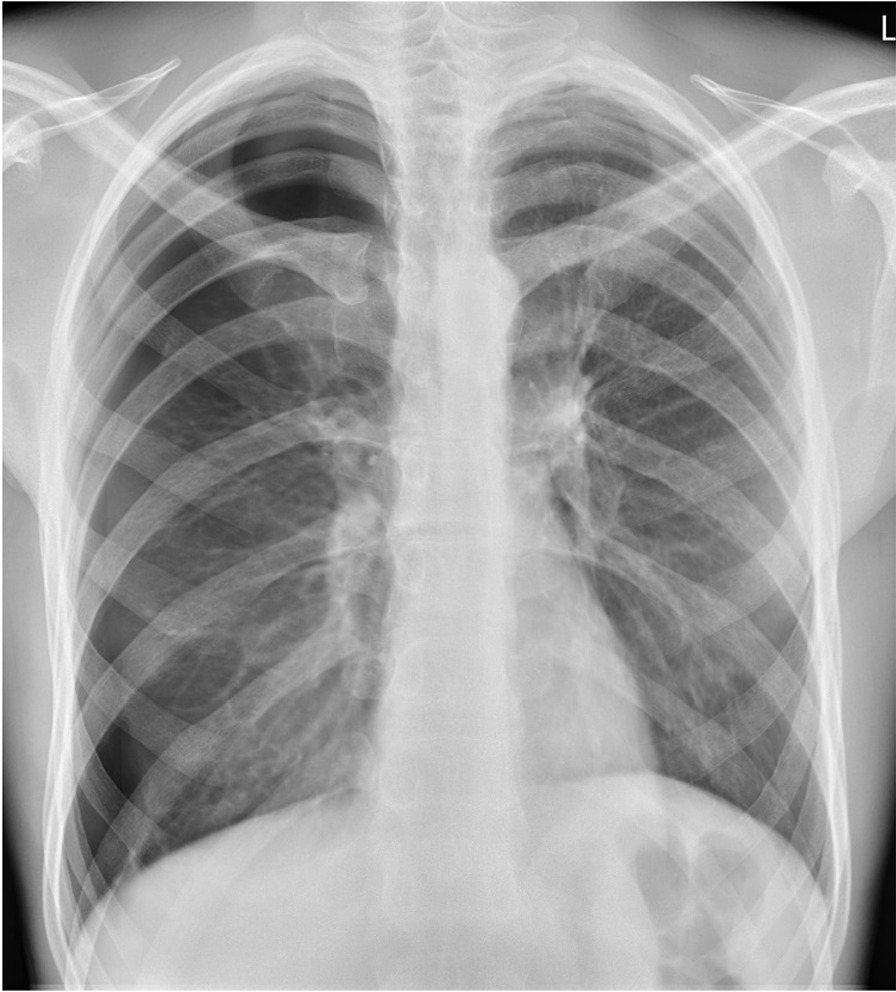
Pneumothorax on right side. No sign of a significant mediastinal shift and thus no evidence of tension pneumothorax. No infiltrate

A African young man of 28 years and a body mass index (BMI) of 19 kg/m^2^ presented for TB screening at Butha-Buthe District Hospital, Lesotho with a productive cough, chest pain, and night sweats starting 3 days prior to presentation. Other exhibited symptoms included fatigue, a sore throat, and rhinorrhea. No fever was reported. He reported no history of TB, previous lung disease, or other comorbidities. On presentation, the patient had a stable cardiorespiratory status, with all vital signs and oxygen saturation within normal range. On clinical examination, there were no signs of edema, dehydration, or enlarged lymph nodes. The SARS-CoV-2 PCR, Xpert MTB/RIF and Xpert MTB/RIF Ultra assays, as well as the TB LAM Ag Test all returned negative. The PA-CXR conducted at Butha-Buthe District Hospital revealed no signs of infiltration or pleural effusion, but a right-sided pneumothorax (Fig. 2; CAD4TB v7 score: 35.54). Hospital admission and intercostal drain placement were recommended as procedure by the study team. The hospital physician, however, opted for a conservative therapy. On the follow-up CXR, the pneumothorax had resolved spontaneously. The patient was in a stable state and was therefore discharged from the hospital.

### Case III

**Fig. 3:**
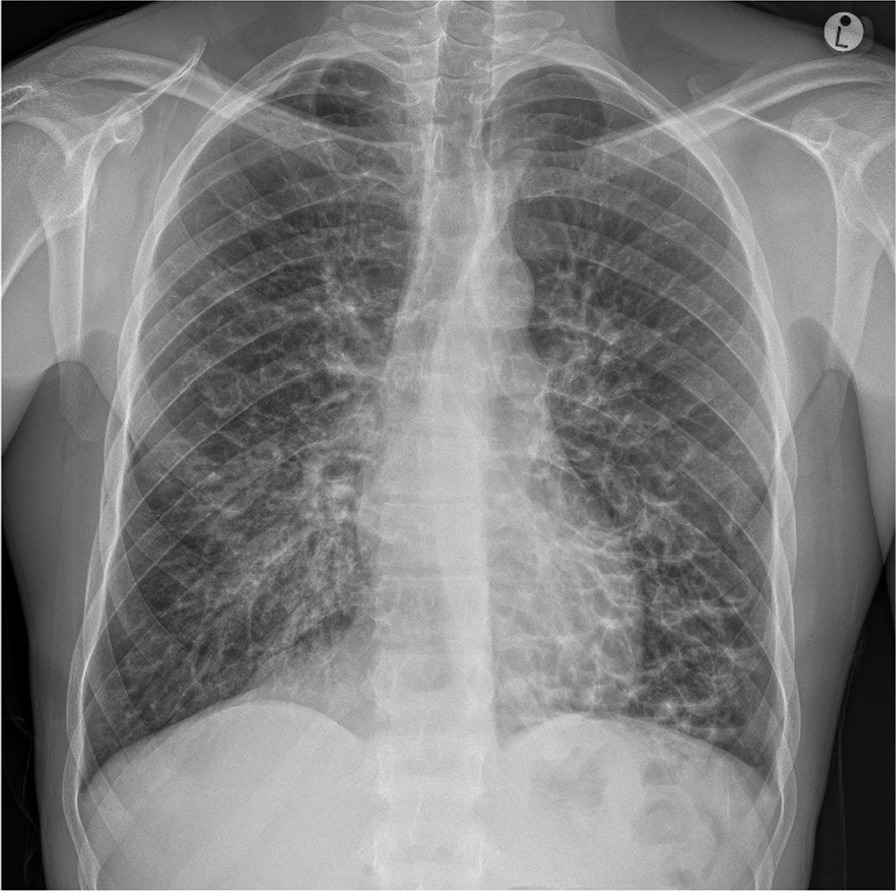
Bilateral massive bronchiectasis with cystic appearance in the middle and lower fields, possibly related to recurrent episodes of bronchopulmonary infection

A 20-year-old African man living with HIV since 2013 presented for TB screening at Caluza Clinic, SA fulfilling all the cardinal symptoms of TB: cough with sputum production, weight loss for a duration of 30 days, fever, and night sweats. The patient also reported chest pain, a sore throat, loss of sense of smell/taste, and rhinorrhea (all symptoms beginning 5 days prior to presentation). Since his HIV diagnosis in 2013, he has been on ART (currently on co-formulated TDF + 3TC + DTG) with good adherence. The last recorded CD4 count and viral load was not available from clinic records. The current VISITECT CD4 count was found to be equal or below 200 cells/µL. He reported a history of two TB episodes, the last episode successfully treated in 2015. Upon clinical examination, no edema, dehydration, pain/discomfort, or enlarged lymph nodes were discovered. Further physical examination was unremarkable. The Xpert MTB/RIF and Xpert MTB/RIF Ultra assays and the TB LAM Ag Test all returned negative. However, the SARS-CoV-2 reverse transcription (RT)-PCR tested positive. A cryptococcal antigen (CrAg) lateral flow assay was also taken and returned negative. The PA-CXR, as interpreted by an expert radiologist at the University Hospital in Basel, revealed massive bronchiectasis with cystic appearance in the right and left middle field, as well as in the right and left lower field, possibly related to recurrent bronchopulmonary infections (Fig. 3; CAD4TB v7 score: 39.36). Upon follow-up 6 months later, the patient reported his general condition since enrollment as improved. He reported intermittent shortness of breath, dry cough, and tiredness. The previous 7 days, however, were symptom-free. Since enrollment in the TB TRIAGE + ACCURACY study, the patient has sought no medical care, and there is no treatment or future care planned.

### Case IV

**Fig. 4:**
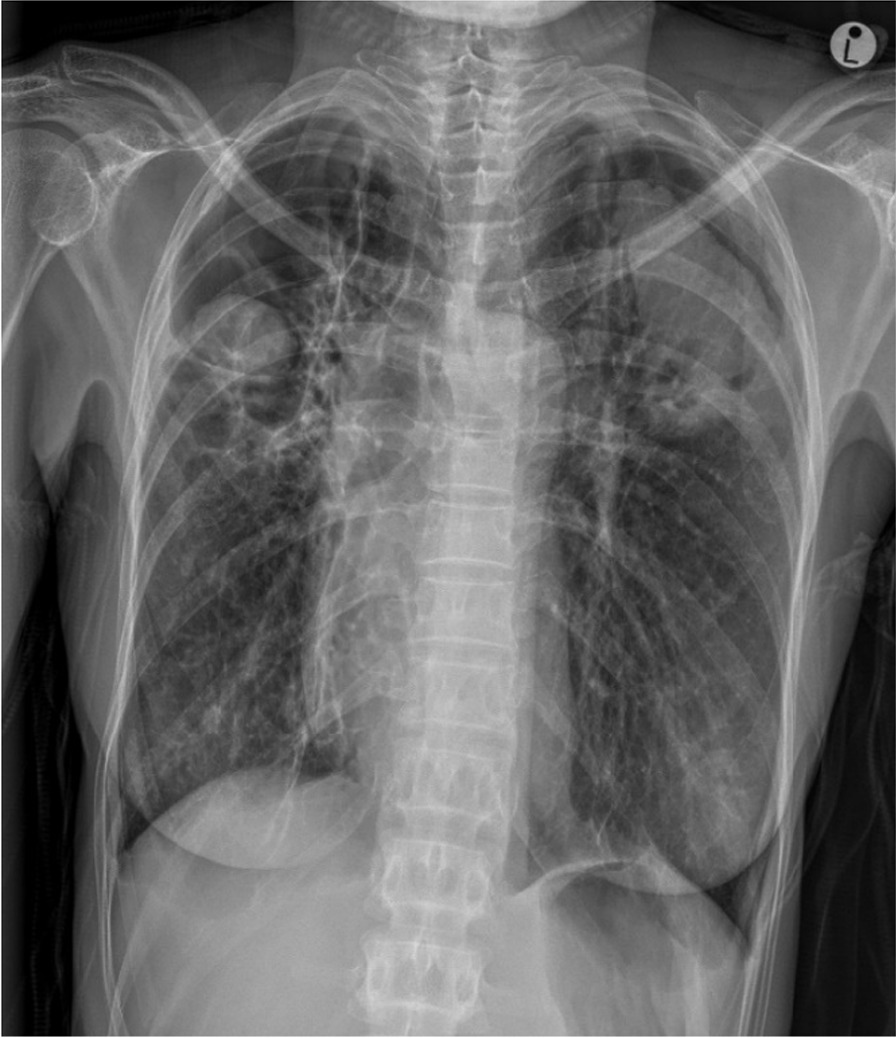
Cavitation in right and left upper fields with solid content, potentially fungus balls/aspergillomas. Bilateral pleural thickening. No sign of infiltrates or pulmonary edema

A 47-year-old African woman living with HIV with a BMI of 14.9 kg/m^2^ reported for TB screening at Caluza Clinic, SA, with a 3-month history of hemoptysis (quantified as a teaspoon full), night sweats, and weight loss. No fever, chest pain, fatigue, or headache was reported. She has been on ART since 2006 with good adherence, on a renal-friendly regimen of abacavir + 3TC + efavirenz due to renal impairment (cause and severity not documented). She has a record of six previous episodes of TB. The last episode was successfully treated in 2012. She also reported a documented history of aspergilloma treated with itraconazole in 2014, and a diagnosis of fibrocavitary lung disease. The participant was last admitted in November 2020 at Harry Gwala Hospital with a primary complaint of hemoptysis. At this admission, she had a computed tomography pulmonary angiogram (CTPA) which showed a bilateral cavitary disease with mycetomas. Upon clinical examination following the enrollment in the TB TRIAGE + ACCURACY study, she was chronically ill in appearance and had temporal wasting, cervical lymphadenopathy, and fingernail clubbing. She was apyrexial and comfortable on room air with SpO_2_ of 98%, and her vital signs were within normal range with a respiration rate of 18 beats per minute. Her chest findings were equal chest expansion, hyperresonance to percussion bilaterally, air entry heard bilaterally but slightly decreased on the right posteriorly, and bilateral amphoric breath sounds with intermittent wheezing. The SARS-CoV-2 PCR and Xpert MTB/RIF and Xpert MTB/RIF Ultra assay results were negative. The TB LAM Ag Test, which detects the LAM antigen in urine, indicating active TB, was positive (1+). The capillary blood sample displayed neutrophilic leukocytosis with neutrophil levels at 76% (normal range 40–60%) and a total leukocyte count of 19.3 × 10^9^/L (normal range 4.0–10.0 × 10^9^/L). The CRP level was elevated at 53 mg/L. CD4 level was tested higher than 200 cells/µL. The PA-CXR revealed cavitation with solid content in the right and left upper field, in addition to bilateral thickening of the pleura, highly suspicious for aspergillomas (Fig. 4; CAD4TB v7 score: 61.7). The patient was called back the following week for an additional sputum and urine Xpert MTB/RIF Ultra, both without detection of *Mycobacterium tuberculosis* (MTB). The patient was then referred to the hospital outpatient clinic where the patient was known for many years.

## Discussion and conclusions

The CAD4TB software has previously been shown to demonstrate high sensitivity for CXR TB-related abnormalities, making it a useful tool for TB detection with version 7 displaying a sensitivity of 88.2% [95% confidence interval (CI) 87.2–89.2%] and specificity of 76% (95% CI 75.1–76.9%), but it is not yet calibrated for the detection of non-TB abnormalities [15, 26]. In all the cases described above, except for the PAVM, the CAD4TBv7 results were above the TB threshold of 28 and thus requiring further testing, but all subsequent TB diagnostic testing was negative. This suggests that an elevated CAD4TB score in the absence of a microbiological diagnosis of TB may indicate a non-TB abnormality and that further referral of these patients should be considered depending on their clinical presentation. Furthermore, in two of the described cases (i.e. PAVM and pneumothorax) the underlying pathology was not detected by the CAD4TB heatmap. The participant with PAVM would therefore have gone undetected by solely analyzing the CXR using the CAD4TB software. Due to the limitation of CAD4TB for non-TB-abnormality identification, the CAD software may miss significant CXR abnormalities that need immediate treatment. PAVM, pneumothorax, massive bronchiectasis, and aspergilloma are all abnormalities requiring medical attention. PAVM can be asymptomatic or symptomatic depending on the degree of the right to left shunt and can cause severe complications including stroke, brain abscess, and hypoxemia [17]. Similarly, failing to correctly treat a pneumothorax can lead to devastating consequences such as progression to a tension pneumothorax, pneumomediastinum, shock, respiratory/cardiac arrest, empyema, or hypoxemic respiratory failure [18]. Depending on the size and the presence or absence of symptoms, a pneumothorax can be treated conservatively through observation, or invasively through chest tube placement. In patients with bronchiectasis, treatment is important to improve symptoms and prevent relapses, as these patients are at a higher risk of developing respiratory infections that require antibiotics. Furthermore, complications such as pneumonia, lung abscess, empyema, septicemia, pulmonary hypertension, or respiratory failure may develop [19]. The detection of aspergillomas, even though often incidental in mild or asymptomatic patients, is important due to the significant risk of evolving complications such as pulmonary fibrosis and hemoptysis that can lead to massive fatal hemorrhage [20]. The treatment of choice in symptomatic patients with aspergilloma is surgical resection. If surgery is not possible, antifungal treatment can be considered [21].

When screening for TB, users of AI software need to be aware that other chest pathologies are likely to be as prevalent as, or more prevalent than, active TB [22]. However, non­-TB CXR abnormalities detected during CXR screening remain poorly characterized in the sub-Saharan African setting, with only minimal literature [22, 23]. There is no structured system for the detection and referral of such patients [24, 25]. In a retrospective analysis of X-ray images from the 2016 Kenya TB Prevalence Survey, a large number of patients with non-TB-related abnormalities were identified, with cardiac and pulmonary diseases accounting for 66% of the non-TB abnormalities in this setting [22]. In 2021, a cross-sectional study in Malawi studying the range of CXR abnormalities during a TB screening reported a high prevalence of non-tuberculous abnormalities found by the CXR-analyzing physician, the most recurrent abnormality being cardiomegaly with 20.7% (95% CI 18.0–23.7%) [23]. In our TB TRIAGE + ACCURACY study [16] among presumptive TB cases, 6.6% of CXR showed non-TB-related CXR abnormalities, as compared with 25.6% of CXR presenting abnormalities categorized as possible TB or highly suspicious for TB.

To improve the detection of non-TB pathologies in TB screening with AI software, it is important to differentiate between health-facility-based screening and community-based screening or prevalence surveys. In a health-facility-based screening setting, such as in our study, the inclusion of an on-site radiologist or radiologically trained individual, if available, would increase the detection of non-TB abnormalities, such as the cases described above. In community-based screening programs or prevalence surveys, where radiological expertise is usually not available, expanding the range of detection in AI software to include non-TB abnormalities would be the best solution to improve the detection of non-TB cases. Even in hospital settings where a radiologist can be consulted, expanding the AI software could prevent the need to involve a radiologist, which is significant considering that there is frequently a lack of radiological expertise in high-burden TB settings [10–12]. Attempts to advance the AI software used in TB detection to include the detection non-TB abnormalities have already been made. A recent study by Qin *et al*., comparing different versions of computer-aided detection products, features the recently updated CAD4TBv7, which allows cardiomegaly analysis [26]. Qure.ai (Qure.ai Technologies Pvt. Ltd, India) has also developed an automatic CXR classifier qXR, which provides indication of TB disease while also detecting other CXR abnormalities such as pleural effusion, nodules/cavities, hilar lymphadenopathy, and atelectasis [27]. Similarly, Annalise CXR V.1.2 (Annalise-AI, Sydney, Australia) has shown promising results, being validated for 124 clinical CXR findings in a multireader, multicase study [28]. It is also the first US Food and Drug Administration (FDA)-cleared model that successfully distinguishes a tension pneumothorax from any pneumothorax [29].

Considering the lack of literature in sub-Saharan Africa concerning non­-TB conditions and abnormalities detected during TB screening, an increase of data collection, characterization of these patients, and the exploration of implications of these diseases for individuals and health systems is needed. This would provide evidence-based requirements to enable the improvement of existing AI software programs and their use in such settings [22]. In the absence of trained radiologists, AI software systems offer an opportunity to perform symptom-agnostic TB screening. This mass implementation of radiography with enhanced AI systems would simultaneously provide the opportunity to combat both respiratory and cardiac diseases and aid in the early detection of treatable non-TB conditions if expanded to the detection of other cardio-pulmonal diseases [22, 30, 31]. Once detected, clear referral pathways, diagnostics, and follow-up plans should ensue [32]. A structural system for the detection and referral of such patients needs to be developed and enforced to ensure medical treatment and care for non-TB cases, such as those described in this series.

In summary, non-TB abnormalities, such as those presented in this case series, are important additional findings detected during TB screening in sub-Saharan Africa. AI software, which is increasingly being used to detect TB on digital X-rays, needs to be further improved and extended to also detect non-TB abnormalities, as there are not enough skilled radiologists in resource-poor settings to analyze X-rays as part of future TB screening.

## Data Availability

The authors of this manuscript are willing to provide additional information regarding the case series.

## Abbreviations

AI: Artificial intelligence
CXR: Chest X-ray
CAD: Computer-aided detection
CRP: C-reactive protein
CrAg: Cryptococcal antigen
CTPA: Computed tomography pulmonary angiogram
DTG: Dolutegravir
LAM: Lipoarabinomannan
MTB: *Mycobacterium tuberculosis*
PA-CXR: Posteroanterior chest X-ray
PAVM: Pulmonary arteriovenous malformation
SA: South Africa
TB: Tuberculosis
TDF: Tenofovir disoproxil fumarate
3TC: Lamivudine
